# Increased cerebellar vermis volume following repetitive transcranial magnetic stimulation in drug-resistant epilepsy: a voxel-based morphometry study

**DOI:** 10.3389/fnins.2024.1421917

**Published:** 2024-10-25

**Authors:** Mingyeong So, Jooheon Kong, Young-Tak Kim, Keun-Tae Kim, Hayom Kim, Jung Bin Kim

**Affiliations:** ^1^Department of Neurology, Korea University Anam Hospital, Korea University College of Medicine, Seoul, Republic of Korea; ^2^Department of Radiology, Massachusetts General Hospital, Harvard Medical School, Boston, MA, United States; ^3^Department of Biomedical Sciences, Korea University College of Medicine, Seoul, Republic of Korea; ^4^Department of Convergence Medicine, Korea University College of Medicine, Seoul, Republic of Korea

**Keywords:** repetitive transcranial magnetic stimulation, voxel-based morphometry, cerebellum, vermis, drug-resistant epilepsy

## Abstract

**Introduction:**

Voxel-based morphometry (VBM) was applied to explore structural changes induced by repetitive transcranial magnetic stimulation (rTMS) and the relationship with clinical outcomes. Moreover, the relationship between each segmented regional gray matter (GM) volume was investigated to identify circuits involved in the rTMS treatment process in patients with drug-resistant epilepsy (DRE).

**Methods:**

Nineteen patients with DRE were finally included in the analysis. A session of rTMS was applied for 5 consecutive days. Participants received either 1,000 or 3,000 pulses, at a frequency of 0.5 Hz and the intensity was set at 90% of the individual’s resting motor threshold. VBM analysis was performed to explore regional GM volume changes 2 months after rTMS application. The regional volume change was correlated with seizure reduction rate. Relationships between changes in GM volume in each anatomically parcellated region were analyzed using a fully-automated segmentation pipeline.

**Results:**

Compared to the baseline, seizure frequency was reduced, and quality of life was improved after rTMS treatment. Regional volume was increased in the cerebellar vermis 2 months after rTMS application. The increased cerebellar vermis volume correlated with the reduced seizure frequency. Regional volume changes in the cerebellar vermis were correlated with changes in the subcortical and cortical GM regions including the thalamus, caudate, and frontal cortex.

**Discussion:**

These results indicate that rTMS treatment effectively reduced seizure frequency in patients with DRE. Increased volume in the cerebellar vermis and activations of the cerebello-thalamo-cortical circuit may be a crucial mechanism underlying the effectiveness of rTMS application in patients with DRE.

## 1 Introduction

Repetitive transcranial magnetic stimulation (rTMS) has been paid attention as a non-invasive treatment alternative for patients with drug-resistant epilepsy (DRE) (Carrette et al., 2016; Fregni et al., 2006; Sun et al., 2012). However, prior findings regarding its therapeutic efficacy are inconsistent (Cooper et al., 2018; Mishra et al., 2020; Tsuboyama et al., 2020), and even studies that have shown effective outcomes could not clearly elucidate the mechanisms underlying mitigating seizure activities, limiting its practical application in clinical settings for treatment of DRE. While it is widely accepted that low-frequency rTMS is beneficial for epilepsy treatment by modulating cortical excitability and reducing synaptic strength via long-term depression (LTD) at the synaptic level (Fregni et al., 2006; Kinoshita et al., 2005; Sun et al., 2012; Tergau et al., 1999; Theodore et al., 2002), the mechanisms of its effectiveness in DRE at a macroscopic neuroanatomical level remain elusive. If a sufficient understanding of the mechanism of rTMS being effective in treating DRE is achieved, clinical usability could be enhanced by optimizing the stimulation protocol, which includes intensity, the number of pulses, and especially target region.

It is well-known that the functioning of the human brain should be understood in the context of its functional connectivity (FC), rather than by the activity of specific regions (Power et al., 2011). Since epilepsy is considered as a network disorder (Spencer, 2002; van Diessen et al., 2013), in order to understand the mechanism of rTMS effect in DRE, a large-scale network approach at the whole brain level may be appropriate. Hence, exploring the anatomical correlates affected by rTMS treatment and identifying the potential circuits connected to those regions could not only help to elucidate the mechanisms involved in the effect for treatment of DRE, but also provide valuable information such as quantitatively evaluating the treatment effect and determining the stimulation target region.

Voxel-based morphometry (VBM) is a fully automated, unbiased, operator-independent magnetic resonance imaging (MRI) analysis technique that is widely used to detect subtle structural differences between groups of subjects, as well as those between before and after treatment (Ashburner and Friston, 2000; Kim et al., 2023). Several lines of evidence indicate that VBM could demonstrate the neuroplastic effects. Changes in gray matter (GM) volume were found in healthy individuals applied rTMS during cognitive tasks and patients with tinnitus following rTMS treatment (Jung and Lambon Ralph, 2016, 2021; Lehner et al., 2014; Poeppl et al., 2018). These findings revealed the potential of rTMS to induce significant structural changes, as well as VBM could be an appropriate tool for identifying the relevant brain regions in the context of neural plasticity.

To the best of our knowledge, there is currently no study investigating brain structural changes following application of rTMS in patients with DRE. In the present study, we applied VBM to explore structural changes affected by rTMS and their relationships with clinical effectiveness such as seizure reduction rate and improved quality of life. In addition, we aimed to investigate the connectivity between regional volume changes found in VBM after rTMS and those in other parcellated GM regions to explore the circuits affected by rTMS in patients with DRE. We hypothesized that the GM volume of specific regions would change after rTMS and be associated with the clinical effectiveness of rTMS in patients with DRE. Our hypothesis was based on the well-established involvement of the thalamocortical tract, as well as other areas implicated in seizure propagation—such as the cerebellum, frontal cortex, and temporal regions—would exhibit changes in GM volume following rTMS. Additionally, we anticipated that the connectivity between these regions, known to play a crucial role in epileptic networks, would reflect shared patterns of volume change.

## 2 Materials and methods

### 2.1 Participants

**FIGURE 1:**
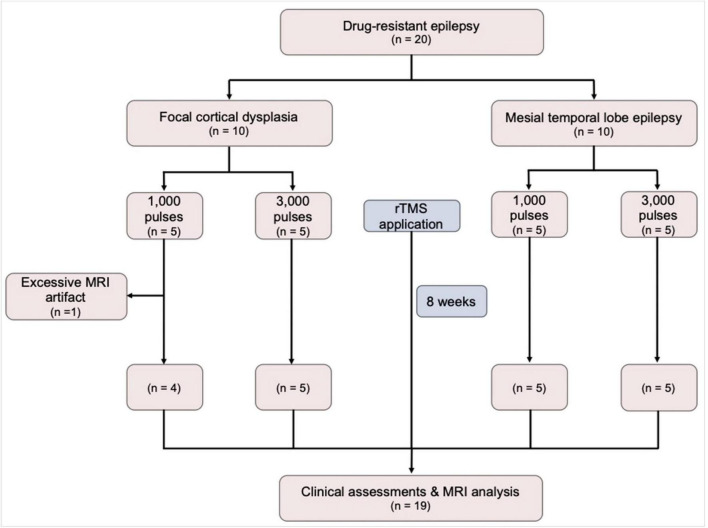
A flow diagram of the study. MRI, magnetic resonance imaging; rTMS, repetitive transcranial magnetic stimulation.

The eligibility criteria for study inclusion were as follows: participants aged 20–70 years, diagnosed with focal drug-resistant epilepsy (DRE) that has not responded to at least two appropriate antiseizure medications (ASMs) without changes in the ASM regimen for a duration of 6 months prior to enrollment in the study. Only DRE patients having electroclinical features and MRI findings indicative of focal cortical dysplasia (FCD) or mesial temporal lobe epilepsy (MTLE) were included. In this study, 10 DRE patients with FCD and 10 with MTLE were prospectively recruited. Within each group of 10 patients, 5 were randomly assigned to receive either 1,000 or 3,000 pulses of rTMS (Figure 1). The required seizure frequency for inclusion was at least one focal to generalized seizure per month and/or two focal seizures per month. The exclusion criteria were as follows: (1) contraindications for rTMS (implanted with intracranial metal devices, pacemakers, or other electrical medical devices); (2) potential for pregnancy or lactating; (3) contraindicated for neuroimaging; (4) psychiatric disorders or currently taking antipsychotic medications; (5) history of cardiovascular diseases, especially arrhythmias, neurological disorder other than epilepsy, or progressive systemic diseases; (6) skull fractures or who had undergone major brain surgery, (7) clinical dementia rating (CDR) score of more than 2 points.

The study followed the ethical guidelines of the Declaration of Helsinki and was approved by the Ministry of Food and Drug Safety of Korea (No. 1251) and the institutional review board of the Korea University Anam Hospital (No. 2021AN0584). Informed consent was obtained from all individual participants included in the study.

### 2.2 Clinical assessments

The primary efficacy outcome of rTMS was the seizure reduction rate at 2 months. The secondary outcomes included changes in quality of life, depressive symptom, and anxiety, which were measured by the Quality of Life in Epilepsy Inventory-10 (QOLIE-10) (Cramer et al., 1996), Beck Depression Inventory (BDI) (Beck et al., 1987), and Hospital Anxiety and Depression Scale (HADS) (Bjelland et al., 2002) at baseline and 2 months following rTMS applications.

### 2.3 Repetitive transcranial magnetic stimulation

**FIGURE 2:**
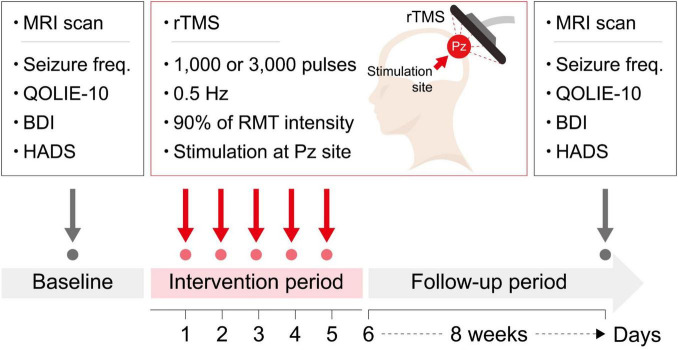
A schematic overview of the study design. Beck Depression Inventory; HADS, Hospital Anxiety and Depression Scale; Seizure freq, seizure frequency; QOLIE-10, Quality of Life in Epilepsy Inventory-10; BDI, RMT, resting motor threshold; rTMS, repetitive transcranial magnetic stimulation.

A session of rTMS was provided for five consecutive days. Participants were seated in specialized chair designed to minimize body movements, ensuring targeted specific stimulation area. Stimulations were delivered to the Pz electrode region following the extended international 10–20 system for electroencephalography electrode placement (Klem et al., 1999), utilizing the ALTMS^®^ device with a figure-eight coil (REMED, Korea). The resting motor evoked potential threshold was determined as the minimum stimulus intensity required to induce contraction of the right thumb at least five times through the stimulation of the left motor cortex ten times. Participants received either 1,000 or 3,000 pulses, with a frequency of 0.5 Hz and an intensity of 90% of the individual’s resting motor threshold. Participants assigned to receive 1,000 pulses were blinded to receiving 2,000 sham stimuli. For sham stimulation, no active stimulation was delivered, while providing simulated stimulator noise of the same frequency (i.e., 0.5 Hz) as for the active stimulation. The rTMS parameters (i.e., stimulation frequency, stimulation intensity, and coil type) were selected based on previous studies, with reference to the most commonly used parameters in clinical trials conducted on patients with DRE. In previous studies, the number of daily pulses ranged widely from 100 to 3,000, with randomized controlled trials typically applying a minimum of 1,000 pulses (Carrette et al., 2016). Based on this prior experience, we selected 1,000 pulses to represent a lower dose and 3,000 pulses to represent a higher dose for the purpose of this study. Additionally, Pz was chosen as the stimulation target area for several reasons. First, in the MTLE group, direct rTMS stimulation of the seizure focus was deemed unfeasible; however, indirect modulation through FC was considered possible by targeting the cortical area. Furthermore, considering the laterality of the seizure focus in MTLE, the cortical area located at the midline was determined to be appropriate for modulating the seizure focus through FC. Second, patients with FCD may have undemonstrated multiple seizure foci or dual pathology. Therefore, it was hypothesized that stimulating a common area with widespread connectivity across all participants could be more efficient than targeting a single site documented as a seizure focus. Third, Pz was selected due to its reflection of posterior cingulate cortex activity, a key node of the default mode network with strong resting-state FC, and its link to the limbic system. Consequently, it was expected that rTMS delivered to this region might be effective for both FCD and MTLE patients. A schematic overview of the study design is shown in Figure 2.

### 2.4 MRI acquisition

MRI scans were acquired using a 3.0 Tesla scanner (Trio whole-body imaging system, Siemens Medical Systems, Iselin, NJ). A T1-weighted magnetization-prepared rapid gradient echo sequence was used (repetition time [TR] = 2300 ms, echo time [TE] = 2.13 ms, field of view = 256 mm, matrix size = 256 × 256; voxel size = 1.0 × 1.0 × 1.0 mm3, and flip angle = 9°) for volumetric analysis.

### 2.5 Voxel-based morphometry

SPM12^1^ was used for data preprocessing and analysis, and VBM was used with DARTEL (Ashburner, 2007). Briefly, the preprocessing included the following steps (Ashburner and Friston, 2000): (1) segmentation, (2) create a template, (3) normalization to Montreal Neurological Institute (MNI) space, and (4) smoothing the modulated GM volume. Detailed procedures are described in our previous works (Kim et al., 2014; Kim et al., 2023).

Differences in the GM volume between the baseline and 2 months after application of rTMS were examined using a paired *t*-test. An absolute GM threshold of 0.2 was used to avoid the possible edge effects around the border between the GM and white matter. Statistical significance was set at a height threshold of *P* < 0.001 and an extent threshold of cluster-level *P* < 0.05, corrected for multiple comparisons using familywise error (FWE). In addition to the overall group analysis before and after rTMS, subgroup analyses were conducted separately for the FCD and MTLE groups, and for the groups that received 1,000 and 3,000 pulses, respectively.

### 2.6 Regional volume connectivity analysis

Fully automated segmentation of the brain was performed using the vol2brain program^2^ (Manjon et al., 2022), which implements a multi-atlas patch-based label fusion segmentation. Single anonymized compressed MRI T1-weighted Nifti files were uploaded through a web interface. Preprocessing consists of de-noising, rough inhomogeneity correction, affine registration to the MNI space, fine inhomogeneity correction, and intensity normalization. To estimate the structural properties of the participants’ brains, a nonlocal intracranial cavity extraction was conducted to create a brain mask. Tissue classification into white matter, GM, and cerebral spinal fluid was conducted on this mask. The pipeline segmented the brain into 116 GM regions including bilateral 9 subcortical and 49 cortical areas (17 frontal, 6 parietal, 8 temporal, 8 occipital, 5 limbic, and 5 insular). In addition, macrostructures including brainstem and cerebellar vermis were segmented. According to the purposes of this study, we were interested in 32 regions as follows: (1) 9 subcortical GM volumes in each side, (2) 6 integrated cortical GM volume, which is the sum of the volumes of each segmented region of the corresponding lobe (i.e., frontal, parietal, temporal, occipital, limbic, and insular cortices) in each side, and (3) brainstem and cerebellar vermis volumes. The connectivity between regions exhibiting volumetric changes post-rTMS, as identified by VBM, and other segmented GM regions was investigated using Pearson’s correlation analysis, with a Bonferroni-corrected significance threshold of *P* < 0.0016.

### 2.7 Statistical analysis

Demographics and clinical characteristics were compared between FCD and MTLE groups, as well as between 1,000 and 3,000 pulses groups using independent *t*-test or χ^2^ test, where appropriate (*P* < 0.05). Clinical variables were compared between the baseline and 2 months after application of rTMS using a paired *t*-test (*P* < 0.05). Voxel values were extracted from the significant cluster in the VBM analysis and were correlated with seizure reduction rate and QOLIE-10 using the Pearson’s correlation (*P* < 0.05).

## 3 Results

Twenty patients with DRE (10 FCD and 10 MTLE) were initially included in this study. Of those, 1 FCD patient who had been received 1,000 pulses was excluded because of excessive artifact, resulting in a total of 19 patients finally included in the analysis. During the application of rTMS, no adverse effects such as headaches, scalp discomfort, seizures, muscle twitching, or burns were observed. Demographics and clinical characteristics are presented in Table 1. The FCD and MTLE groups did not differ in the age, sex, number of ASMs, MMSE, the proportion of hypertension, diabetes, dyslipidemia, alcohol drinkers, and smokers. There were no differences in all demographic and clinical characteristics between patients who received 1,000 pulses and those who received 3,000 pulses.

**TABLE 1:** Demographics and clinical characteristics.

	FCD (*n* = 9)	MTLE (*n* = 10)	*P*-value	1,000 pulses (*n* = 9)	3,000 pulses (*n* = 10)	*P*-value
Age, years (SD)	45.11 (15.81)	49.10 (11.52)	0.535	47.11 (10.89)	47.30 (16.06)	0.977
Women, *n* (%)	6 (66.7)	9 (90.0)	0.303	6 (66.7)	9 (90.0)	0.303
Education, years (SD)	11.44 (2.70)	10.90 (4.61)	0.761	12.67 (3.64)	9.80 (3.42)	0.095
Number of antiseizure medications (SD)	3.44 (1.24)	3.90 (1.37)	0.459	3.67 (0.87)	3.70 (1.64)	0.956
Mini-mental status examination (SD)	25.50 (3.50)	26.70 (3.20)	0.459	27.00 (3.28)	25.33 (3.28)	0.297
Hypertension, *n* (%)	2 (22.2)	0 (0.0)	0.211	1 (11.1)	1 (10.0)	1.000
Diabetes, *n* (%)	0 (0.0)	0 (0.0)	NA	0 (0.0)	0 (0.0)	NA
Dyslipidemia, *n* (%)	2 (22.2)	1 (10.0)	0.582	1 (11.1)	2 (20.0)	1.000
Alcohol drinking, *n* (%)	1 (11.1)	1 (10.0)	1.000	2 (22.2)	0 (0.0)	0.211
Smoking, *n* (%)	1 (11.1)	0 (0.0)	0.474	0 (0.0)	1 (10.0)	1.000

FCD, focal cortical dysplasia; MTLE, mesial temporal lobe epilepsy.

**FIGURE 3:**
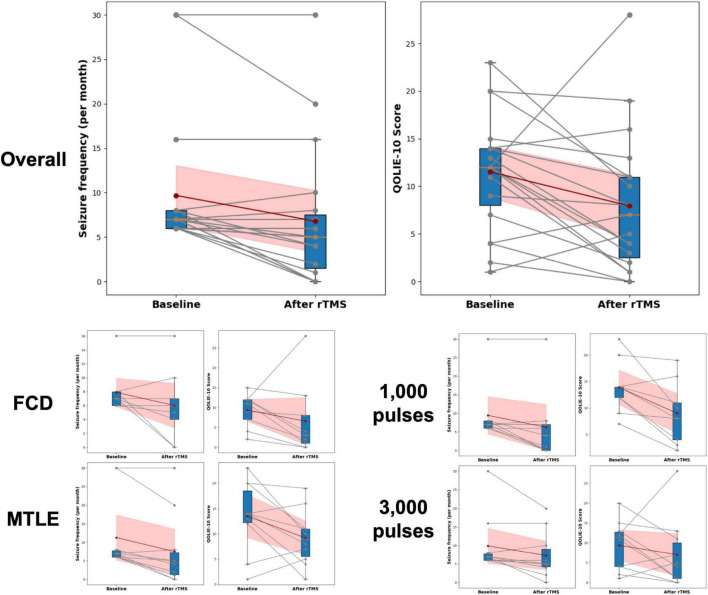
Changes in seizure frequency and Quality of Life in Epilepsy Inventory-10 (QOLIE-10) following rTMS. Changes in seizure frequency and QOLIE-10 before and after rTMS treatment are presented. The box plots summarize the distribution of scores at each time point, indicating median, interquartile ranges, and outliers. Individual changes in seizure frequency and QOLIE-10 scores for each participant are shown with gray lines connecting the baseline and post-treatment (After rTMS) values, with corresponding dots marking the actual scores. The red dots represent the mean seizure frequency and QOLIE-10 scores at baseline and after treatment, connected by a dark red line to highlight the average trend in score changes. The shaded red area indicates the 95% Confidence Interval (CI) around the mean changes. FCD, focal cortical dysplasia; MTLE, mesial temporal lobe epilepsy; QOLIE-10, Quality of Life in Epilepsy Inventory-10.

Clinical outcomes for rTMS application are presented in Table 2 and Figure 3. Compared with the baseline, the seizure frequency was reduced 2 months after application of rTMS (*P* < 0.001). Quality of life measured by QOLIE-10 was improved after rTMS treatment, relative to the baseline (*P* = 0.033). The BDI, HADS-anxiety, and HADS-depression scores were not changed after application of rTMS.

**TABLE 2:** Clinical outcome following rTMS.

FCD				MTLE			
**Outcome measurements**	**Baseline**	**After rTMS**	***P*-value**	**Outcome measurements**	**Baseline**	**After rTMS**	***P*-value**
Seizure frequency (per month)	7.89 (3.14)	6.00 (4.92)	0.029	Seizure frequency (per month)	11.30 (9.88)	7.50 (9.85)	0.006
QOLIE-10	9.33 (4.24)	6.56 (9.08)	0.148	QOLIE-10	13.50 (6.87)	9.20 (5.45)	0.055
BDI	10.00 (8.97)	9.22 (9.50)	0.785	BDI	12.40 (10.96)	15.50 (9.38)	0.297
HADS-anxiety	5.00 (3.16)	5.33 (4.47)	0.843	HADS-anxiety	5.90 (4.46)	6.70 (3.62)	0.423
HADS-depression	6.33 (3.64)	7.11 (5.08)	0.648	HADS-depression	8.80 (4.80)	9.80 (3.33)	0.591
**1,000 pulses**				**3,000 pulses**			
**Outcome measurements**	**Baseline**	**After rTMS**	***P*-value**	**Outcome measurements**	**Baseline**	**After rTMS**	***P*-value**
Seizure frequency (per month)	9.44 (7.75)	6.22 (9.47)	0.024	Seizure frequency (per month)	9.90 (7.68)	7.30 (6.27)	0.048
QOLIE-10	13.89 (9.47)	9.00 (5.77)	0.014	QOLIE-10	9.40 (6.29)	7.00 (8.67)	0.388
BDI	11.33 (7.63)	15.00 (10.61)	0.246	BDI	11.20 (11.95)	10.30 (8.79)	0.738
HADS-anxiety	6.33 (3.20)	6.22 (3.23)	0.919	HADS-anxiety	4.70 (4.32)	5.90 (4.75)	0.425
HADS-depression	8.67 (2.06)	9.22 (4.26)	0.759	HADS-depression	6.70 (5.68)	7.90 (4.56)	0.499

BDI, Beck Depression Inventory; FCD, focal cortical dysplasia; HADS, Hospital Anxiety and Depression Scale; MTLE, mesial temporal lobe epilepsy; QOLIE-10, Quality of Life in Epilepsy Inventory-10; rTMS, repetitive transcranial magnetic stimulation.

**FIGURE 4:**
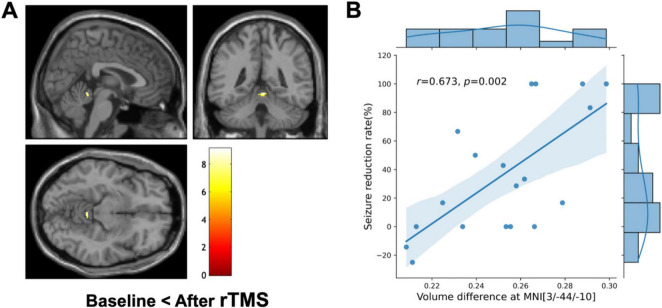
Voxel-based morphometry (VBM) and clinical correlation. **(A)** VBM shows a significant regional increase in the cerebellar vermis (MNI coordinate = 3/–44/-10, cluster-level FWE-corrected *P* = 0.015) after repetitive transcranial magnetic stimulation (rTMS) compared to the baseline. The color bar represents the *T*-value. The left side of each picture is the left side of the brain. **(B)** Combined scatterplot and boxplot depicting the relationship between volume difference at cerebellar vermis (MNI coordinates: 3/–44/–10) and the seizure reduction rate. Each data point represents an individual participant. The amount of regional volume increases in cerebellar vermis is positively correlated with seizure reduction rate (Pearson’s *r* = 0.673, *P* = 0.002). The side boxplot provides a summary distribution of the seizure reduction rates corresponding to the volume differences observed.

VBM showed a significant increase in regional GM volume in the cerebellar vermis (MNI coordinates of local maxima = 3/−44/-10, extent threshold k = 30 voxels, cluster-level FWE-corrected *P* = 0.015, Figure 4A). No area of decreased GM volume was found after rTMS at the same threshold. The amount of regional volume increases in cerebellar vermis was positively correlated with seizure reduction rate (*r* = 0.673, *P* = 0.002, Figure 4B). The volume changes in cerebellar vermis were not correlated with the QOLIE-10, BDI, HADS-anxiety, and HADS-depression scores. No GM volume changes were observed in the subgroup analyses conducted based on each etiology (i.e., FCD and MTLE) and the number of stimulation pulses (i.e., 1,000 and 3,000) at the same threshold.

**FIGURE 5:**
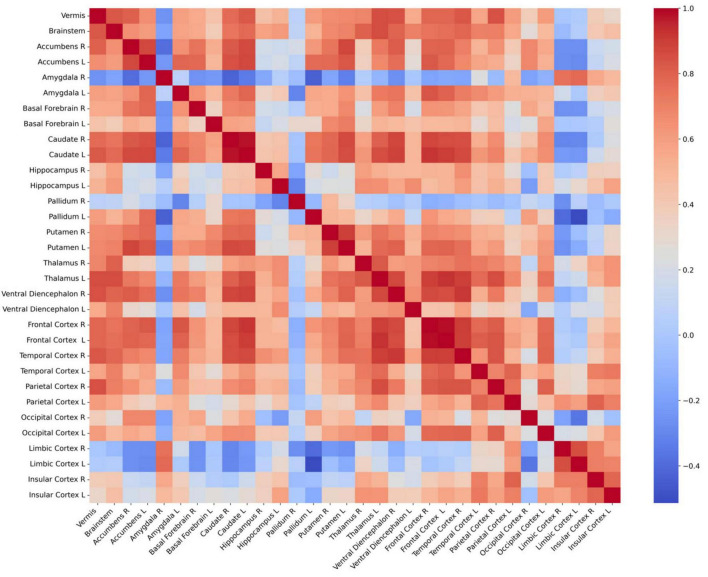
Connectivity matrix between 32 regional volume changes. Each cell in the heatmap of correlation matrix represents the Pearson’s correlation coefficient between the volumes of two brain regions. Red denotes positive correlations, blue denotes negative correlations, and white denotes neutral correlations. L, left; R, right.

The connectivity of volume changes between the 32 GM regions is presented in Figure 5. Significant correlations were found between volume changes in the cerebellar vermis and those in the bilateral thalamus, caudate, frontal cortices, right ventral diencephalon, parietal and temporal cortices, and accumbens, as well as the left putamen and brainstem (*P* < 0.0016). Results of correlation analyses are detailed in Table 3. In the subgroup analyses, the MTLE group showed volume changes in the bilateral thalamus that correlated with those in the vermis. In the FCD group, regions correlated with volume changes in the vermis included the brainstem, temporal cortex, parietal cortex, and left thalamus. When 1,000 pulses were applied, volume changes in the right ventral diencephalon correlated with those in the vermis, whereas in the group that received 3,000 pulses, volume changes in the left thalamus, right temporal cortex, brainstem, and left caudate correlated with vermis volume changes. Results of subgroup analyses are presented in Supplementary Figure 1 and Supplementary Tables 1, 2.

**TABLE 3:** Correlation analysis between volume changes in cerebellar vermis and other gray matter regions.

Regions	Laterality	Pearson’s rho	*P*-value
Thalamus	L	0.859957	**0.000002**
Ventral diencephalon	R	0.850665	**0.000004**
Parietal cortex	R	0.847898	**0.000005**
Temporal cortex	R	0.832361	**0.000010**
Brainstem	-	0.829929	**0.000011**
Caudate	L	0.824634	**0.000014**
Accumbens	R	0.806231	**0.000031**
Frontal cortex	R	0.788191	**0.000061**
Frontal cortex	L	0.788076	**0.000061**
Caudate	R	0.770773	**0.000112**
Thalamus	R	0.687362	**0.001147**
Putamen	L	0.680551	**0.001342**
Putamen	R	0.663134	0.001969
Accumbens	L	0.659759	0.002116
Occipital cortex	L	0.615310	0.005044
Parietal cortex	L	0.610985	0.005453
Pallidum	L	0.604463	0.006119
Amygdala	L	0.593354	0.007407
Temporal cortex	L	0.569967	0.010841
Basal forebrain	R	0.548170	0.015101
Ventral diencephalon	L	0.546885	0.015388
Hippocampus	L	0.516099	0.023693
Hippocampus	R	0.454225	0.050744
Basal forebrain	L	0.442558	0.057770
Insular cortex	R	0.385516	0.103086
Occipital cortex	R	0.383489	0.105066
Insular cortex	L	0.320936	0.180337
Amygdala	R	−0.255962	0.290185
Pallidum	R	0.077314	0.753069
Limbic cortex	L	0.018814	0.939064
Limbic cortex	R	−0.005000	0.983793

Bold font denotes statistical significance (Bonferroni-corrected *P* < 0.0016). L, left; R, right.

## 4 Discussion

We investigated the acute effects of rTMS on GM volumes in patients with DRE. The major findings were as follows: (1) regional volume was increased in the cerebellar vermis 2 months after rTMS application, (2) the increased volume in the cerebellar vermis was correlated with reduced seizure frequency, and (3) regional volume changes in the cerebellar vermis were correlated with those in subcortical and cortical GM regions including thalamus, caudate, and frontal cortex.

The cerebellum, traditionally known to be associated with motor functions, has been increasingly recognized for its involvement in the role in the pathophysiological mechanisms underlying epilepsy (Kim et al., 2019; Krook-Magnuson et al., 2014; Stieve et al., 2023; Streng et al., 2023; Streng and Krook-Magnuson, 2021; Wang et al., 2023; Zhou et al., 2021). The vulnerability of the cerebellum in epilepsy is a well-established phenomenon supported by a variety of studies highlighting its susceptibility to injury, neurodegeneration, excitotoxic damage, neuron loss, and oxidative stress in the context of seizure disorder (Froula et al., 2023; Kerestes et al., 2024; Lawson et al., 2000; Lomoio et al., 2011; Lores Arnaiz et al., 1998; Streng et al., 2023; Streng and Krook-Magnuson, 2021). These cerebellum’s susceptibility to damage could be implicated in the mechanisms underlying seizure initiation and epileptogenesis. Moreover, the vulnerability can potentially contribute to comorbidities and negative outcomes in epilepsy, such as cognitive impairments, altered regulatory processes, and sudden unexpected death in epilepsy (SUDEP) (Hellwig et al., 2013; Wang et al., 2023), as damage to the cerebellum can further impair the neurological function in patients with epilepsy. These observations have spurred interest in the cerebellum as a potential target for novel therapeutic interventions aimed at mitigating the burdens of epilepsy. Indeed, studies employing cerebellar neuromodulation have shown promising results, including reduced seizure frequency and severity in some patients with DRE (Krook-Magnuson et al., 2014; Piper et al., 2022; Stieve et al., 2023; Streng and Krook-Magnuson, 2021). These findings not only validated the cerebellum’s role in epilepsy but also highlighted the potential of cerebellar-targeted interventions to enhance treatment outcomes for patients. Taken together, the cerebellum has been implicated in the pathogenesis of seizure onset as well as a variety of complications in patients with epilepsy, making it a potential target for neuromodulations. Notably, we found that the volume changes in left thalamus exhibited significant correlations with those in vermis in most individual groups and in the overall group. These results suggest that the cerebellar vermis and thalamus are involved in the epileptic network regardless of etiology. This finding further supports our hypothesis that the cerebello-thalamo-cortical network may serve as a key target for effective rTMS treatment aimed at reducing seizures.

An increase volume found in VBM usually is usually understood as increased function in the corresponding area (Kim et al., 2023). Therefore, we speculate that our findings of increased volume in the cerebellar vermis after rTMS treatment could be interpreted as enhanced function of the cerebellar vermis area in mediating the pathological condition of DRE patients through rTMS-induced activations of inhibitory interneurons. Several lines of evidence indicate that neural plasticity could be induced by various stimulations, even by cognitive tasks (Jung and Lambon Ralph, 2016, 2021; Lehner et al., 2014; May et al., 2007; Poeppl et al., 2018). Volumetric analysis of MRI has demonstrated the structural changes in association with neural plasticity, sensitively (Jung and Lambon Ralph, 2021; Lehner et al., 2014; May et al., 2007; Poeppl et al., 2018). Given the interpretability of structural changes in the perspective of neural plasticity, our findings of volume increases in the cerebellar vermis and their relationship with reduced seizure frequency could be considered as consequences of therapeutic effects by rTMS-induced neural plasticity in patients with DRE.

It is unclear why rTMS applied to the parietal cortex at the Pz electrode attachment location in patients with DRE resulted in changes in the volume of the cerebellar vermis region away from the direct stimulation location. However, this can be explained by the knowledge that the cerebellum has multiple synapses and interconnections with a very large number of cortical and subcortical areas (Kang et al., 2021; Novello et al., 2024), suggesting that the cerebellum can be influenced through circuits connected to it, even if no direct stimulation to the cerebellum is given. Among the wide range of regions connected to the cerebellum and its circuits, our connectivity analysis of regional volume changes showed that volume changes in the cerebellar vermis following rTMS were particularly connected to those in the thalamus, caudate, frontal cortex, and brainstem. These findings are in line with previous observations that cerebellum was connected with thalamus, brainstem, and diencephalon (Novello et al., 2024), and that cerebellar stimulation could mitigate seizure activity via cerebello-thalamo-cortical pathway in patients with DRE (Eelkman Rooda et al., 2021; Piper et al., 2022). Our results suggest that stimulation of cerebellar vermis regions may be effective in the application of rTMS in patients with DRE, and that the cerebello-thalamo-cortical pathway may be the corresponding circuit that allows stimulation to produce therapeutic effects.

To the best of our knowledge, this is the first study to investigate the effect of rTMS in DRE patients in terms of neural plasticity through VBM analysis. Our study controlled the effect of etiology and seizure focus by equally assigning the number of patients with FCD and MTLE. In addition, we evaluated the effect of rTMS and analyzed the change in GM volume after rTMS by controlling the difference between 1,000 and 3,000 pulses. Therefore, our results can be interpreted as generalizable findings that are not affected by etiology, seizure focus, or the number of pulses.

There are several limitations to our study. First, the sample size was relatively small, making our imaging findings less robust, and likely limiting our ability to detect fine structural changes in response to rTMS within each group (i.e., FCD and MTLE). With a more sufficiently powered sample size, it is plausible that distinct regions might exhibit significant structural changes in response to rTMS within each group. Future studies with larger cohorts are needed to further explore these potential differences and to confirm the findings of our study. Second, we only observed brain volume changes from baseline to 2 months later, and there is no information about serial changes and long-term effects until the rTMS effect is washed out. Additional longitudinal follow-up studies are needed to analyze long-term effects and related brain volume change patterns. Third, the interpretation of the volume connectivity results in the cerebello-thalamo-cortical region as functionally connected has an inherent limitation in the assumption. Finally, since there is no fully sham-controlled group, there are limitations in determining whether the results of VBM and regional volume change connectivity were influenced only by rTMS.

## 5 Conclusion

Our results indicate that rTMS treatment was effective for reducing seizure frequency in patients with DRE. Cerebellar vermis volume was increased 2 months after rTMS, and the increased volume was related to seizure reduction rate. In addition, volume changes in the cerebellar vermis were connected to those in the thalamus and frontal cortex. Our findings suggest that increased volume in the cerebellar vermis and activations of the cerebello-thalamo-cortical circuit may be a crucial mechanism underlying effectiveness of rTMS application in patients with DRE.

## Data Availability

The data analyzed in this study is subject to the following licenses/restrictions: The datasets used and/or analyzed during this study are available from the corresponding author upon reasonable request. Requests to access these datasets should be directed to Jung Bin Kim, kjbin80@korea.ac.kr.

## Footnotes

1
http://www.fil.ion.ucl.ac.uk/spm

2
https://www.volbrain.net/services/vol2Brain
