# Supplementary material for: Increased cerebellar vermis volume following repetitive transcranial magnetic stimulation in drug-resistant epilepsy: a voxel-based morphometry study
Source: Front Neurosci. 2024 Oct 25;18:1421917. doi: 10.3389/fnins.2024.1421917 (PMC11544559; doi:10.3389/fnins.2024.1421917)
Supplement: Supplementary file 1 [file Data_Sheet_1.docx]

**SUPPLEMENTARY TABLE 1** Correlation analysis of volume changes between the cerebellar vermis and other gray matter regions in each etiologic group.

| **FCD** | | | | **MTLE** | | | |
| --- | --- | --- | --- | --- | --- | --- | --- |
| **Regions** | **Laterality** | **Pearson's rho** | ***P* value** | **Regions** | **Laterality** | **Pearson's rho** | ***P* value** |
| Brainstem | - | 0.948332 | **0.000098** | Thalamus | L | 0.929468 | **0.000099** |
| Temporal Cortex | R | 0.918486 | **0.000470** | Thalamus | R | 0.890020 | **0.000559** |
| Parietal Cortex | R | 0.914208 | **0.000560** | Frontal Cortex | L | 0.840546 | 0.002322 |
| Thalamus | L | 0.898453 | **0.000994** | Frontal Cortex | R | 0.792934 | 0.006212 |
| Ventral Diencephalon | R | 0.868687 | 0.002370 | Caudate | L | 0.789980 | 0.006549 |
| Accumbens | R | 0.860352 | 0.002915 | Brainstem | - | 0.769702 | 0.009221 |
| Caudate | L | 0.826724 | 0.005992 | Ventral Diencephalon | R | 0.767146 | 0.009605 |
| Caudate | R | 0.803440 | 0.009093 | Insular Cortex | L | 0.740601 | 0.014284 |
| Frontal Cortex | R | 0.783668 | 0.012457 | Parietal Cortex | R | 0.736894 | 0.015044 |
| Thalamus | R | 0.781672 | 0.012837 | Temporal Cortex | L | 0.719522 | 0.018985 |
| Frontal Cortex | L | 0.776894 | 0.013777 | Insular Cortex | R | 0.693488 | 0.026147 |
| Pallidum | L | 0.754616 | 0.018775 | Putamen | R | 0.656693 | 0.039142 |
| Putamen | R | 0.742528 | 0.021932 | Temporal Cortex | R | 0.652501 | 0.040854 |
| Amygdala | L | 0.741265 | 0.022280 | Parietal Cortex | L | 0.641922 | 0.045395 |
| Basal Forebrain | L | 0.713962 | 0.030736 | Occipital Cortex | L | 0.608707 | 0.061817 |
| Accumbens | L | 0.710040 | 0.032100 | Putamen | L | 0.592854 | 0.070869 |
| Putamen | L | 0.702025 | 0.035009 | Limbic Cortex | L | 0.566819 | 0.087530 |
| Parietal Cortex | L | 0.693351 | 0.038344 | Hippocampus | L | 0.551060 | 0.098737 |
| Occipital Cortex | L | 0.690414 | 0.039517 | Ventral Diencephalon | L | 0.531768 | 0.113644 |
| Hippocampus | R | 0.667466 | 0.049494 | Caudate | R | 0.502485 | 0.138832 |
| Ventral Diencephalon | L | 0.652519 | 0.056782 | Hippocampus | R | 0.494369 | 0.146368 |
| Temporal Cortex | L | 0.637690 | 0.064651 | Accumbens | R | 0.491588 | 0.149007 |
| Hippocampus | L | 0.577646 | 0.103333 | Pallidum | R | 0.416346 | 0.231375 |
| Basal Forebrain | R | 0.567333 | 0.111118 | Pallidum | L | -0.415849 | 0.231989 |
| Amygdala | R | -0.531892 | 0.140502 | Accumbens | L | 0.259082 | 0.469793 |
| Occipital Cortex | R | 0.490945 | 0.179593 | Limbic Cortex | R | 0.246020 | 0.493238 |
| Insular Cortex | R | 0.367230 | 0.330940 | Basal Forebrain | R | 0.240529 | 0.503241 |
| Limbic Cortex | L | -0.280274 | 0.465099 | Amygdala | R | 0.174999 | 0.628700 |
| Pallidum | R | -0.269141 | 0.483738 | Basal Forebrain | L | 0.172097 | 0.634491 |
| Limbic Cortex | R | -0.112271 | 0.773672 | Amygdala | L | 0.145488 | 0.688396 |
| Insular Cortex | L | 0.094824 | 0.808267 | Occipital Cortex | R | 0.103984 | 0.774978 |

Bold font denotes statistical significance (Bonferroni-corrected *P* < 0.0016). FCD, focal cortical dysplasia; L, left; R, right; MTLE, mesial temporal lobe epilepsy.

**SUPPLEMENTARY TABLE 2** Correlation analysis of volume changes between the cerebellar vermis and other gray matter regions according to the number of applied pulses.

| **1,000 pulses** | | | | **3,000 pulses** | | | |
| --- | --- | --- | --- | --- | --- | --- | --- |
| **Regions** | **Laterality** | **Pearson's rho** | ***P* value** | **Regions** | **Laterality** | **Pearson's rho** | ***P* value** |
| Ventral Diencephalon | R | 0.884952 | **0.001517** | Thalamus | L | 0.925279 | **0.000125** |
| Brainstem | - | 0.855502 | 0.003269 | Temporal Cortex | R | 0.902275 | **0.000354** |
| Thalamus | L | 0.828523 | 0.005788 | Brainstem | - | 0.884773 | **0.000670** |
| Parietal Cortex | R | 0.793219 | 0.010743 | Caudate | L | 0.864774 | **0.001239** |
| Insular Cortex | R | 0.739902 | 0.022661 | Ventral Diencephalon | R | 0.855102 | 0.001613 |
| Hippocampus | L | 0.720417 | 0.028575 | Parietal Cortex | R | 0.852276 | 0.001736 |
| Insular Cortex | L | 0.694455 | 0.037908 | Caudate | R | 0.843404 | 0.002168 |
| Thalamus | R | 0.692604 | 0.038640 | Accumbens | R | 0.830353 | 0.002937 |
| Hippocampus | R | 0.690111 | 0.039640 | Frontal Cortex | R | 0.825529 | 0.003265 |
| Temporal Cortex | R | 0.687468 | 0.040718 | Frontal Cortex | L | 0.814928 | 0.004078 |
| Frontal Cortex | L | 0.653193 | 0.056439 | Thalamus | R | 0.800081 | 0.005448 |
| Temporal Cortex | L | 0.636541 | 0.065287 | Pallidum | L | 0.742570 | 0.013892 |
| Accumbens | R | 0.601006 | 0.086953 | Putamen | R | 0.716539 | 0.019727 |
| Ventral Diencephalon | L | 0.593362 | 0.092123 | Parietal Cortex | L | 0.715701 | 0.019939 |
| Pallidum | R | 0.583739 | 0.098894 | Amygdala | L | 0.715040 | 0.020107 |
| Frontal Cortex | R | 0.565592 | 0.112466 | Occipital Cortex | L | 0.699666 | 0.024303 |
| Putamen | L | 0.549406 | 0.125469 | Accumbens | L | 0.690390 | 0.027107 |
| Caudate | L | 0.535440 | 0.137375 | Putamen | L | 0.686949 | 0.028201 |
| Parietal Cortex | L | 0.525058 | 0.146641 | Basal Forebrain | L | 0.648246 | 0.042642 |
| Limbic Cortex | L | 0.501286 | 0.169197 | Basal Forebrain | R | 0.635425 | 0.048346 |
| Putamen | R | 0.473535 | 0.197892 | Ventral Diencephalon | L | 0.570874 | 0.084785 |
| Occipital Cortex | L | 0.356921 | 0.345720 | Temporal Cortex | L | 0.557694 | 0.093914 |
| Basal Forebrain | R | 0.320473 | 0.400463 | Occipital Cortex | R | 0.547311 | 0.101531 |
| Accumbens | L | 0.296407 | 0.438644 | Amygdala | R | -0.510535 | 0.131596 |
| Pallidum | L | -0.293455 | 0.443434 | Hippocampus | L | 0.497926 | 0.143035 |
| Caudate | R | 0.282855 | 0.460821 | Hippocampus | R | 0.370188 | 0.292359 |
| Amygdala | L | 0.133123 | 0.732777 | Limbic Cortex | L | -0.269440 | 0.451558 |
| Amygdala | R | 0.104154 | 0.789729 | Insular Cortex | R | 0.215115 | 0.550613 |
| Occipital Cortex | R | -0.086149 | 0.825580 | Limbic Cortex | R | -0.085961 | 0.813344 |
| Limbic Cortex | R | 0.078127 | 0.841649 | Insular Cortex | L | 0.051101 | 0.888508 |
| Basal Forebrain | L | 0.074495 | 0.848940 | Pallidum | R | -0.049085 | 0.892886 |

Bold font denotes statistical significance (Bonferroni-corrected *P* < 0.0016). FCD, focal cortical dysplasia; L, left; R, right; MTLE, mesial temporal lobe epilepsy.


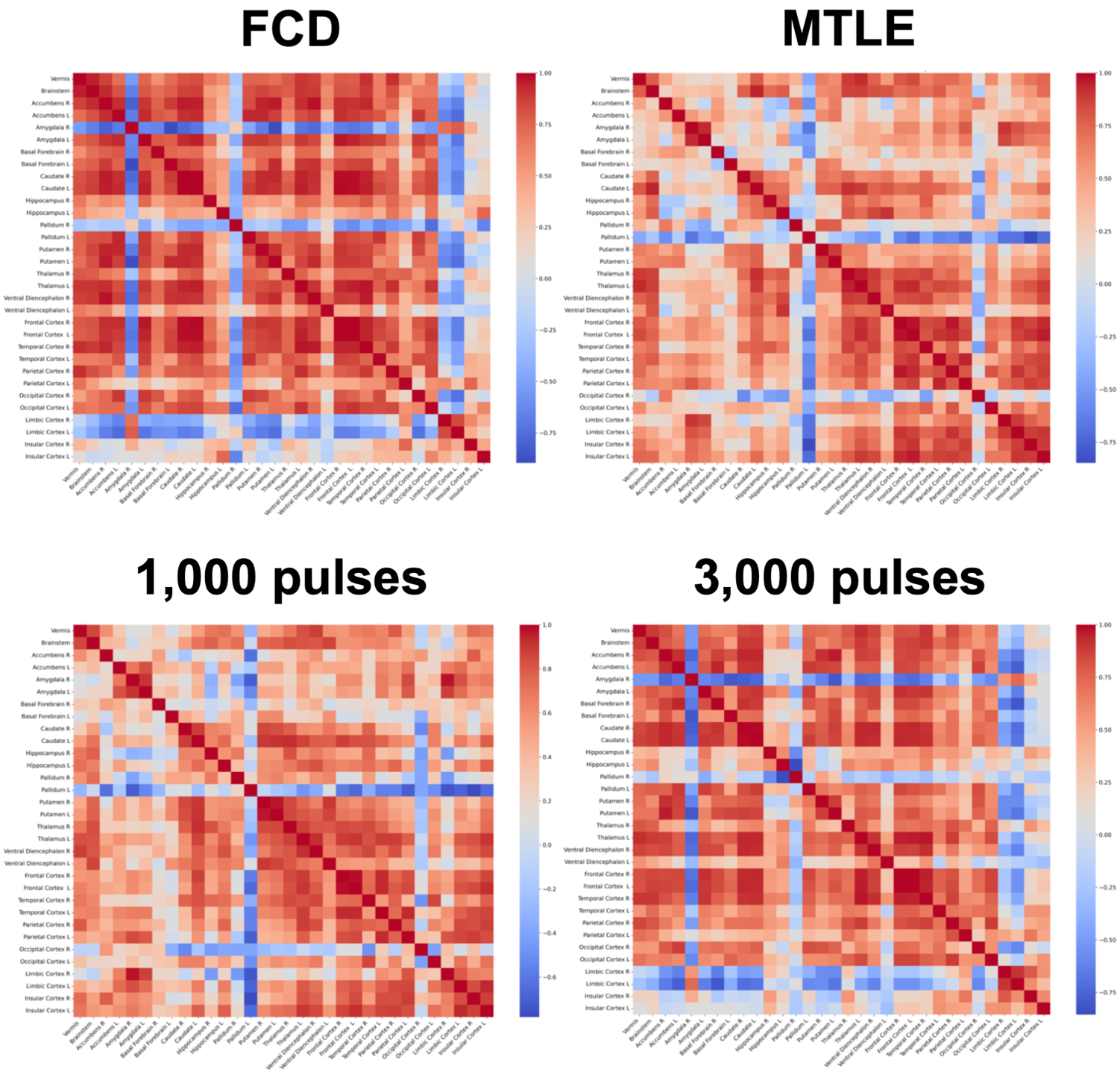


**SUPPLEMENTARY FIGURE** Connectivity matrix between 32 regional volume changes. Each cell in the heatmap of correlation matrix represents the Pearson’s correlation coefficient between the volumes of two brain regions. Red denotes positive correlations, blue denotes negative correlations, and white denotes neutral correlations. FCD, focal cortical dysplasia; MTLE, mesial temporal lobe epilepsy; L, left; R, right.
